# Resource use and cost associated with computerized decision support system and usual care in managing patients with atrial fibrillation: analysis of IMPACT-AF randomized trial data

**DOI:** 10.1186/s12911-023-02329-7

**Published:** 2023-10-18

**Authors:** Brittany Humphries, Jafna L Cox, Ratika Parkash, Lehana Thabane, Gary A Foster, James MacKillop, Joanna Nemis-White, Laura Hamilton, Antonio Ciaccia, Shurjeel H Choudhri, Bruno Kovic, Feng Xie

**Affiliations:** 1 Department of Health Research Methods, Evidence and Impact, McMaster University, Hamilton, ON Canada; 2 Division of Cardiology, Department of Medicine, Dalhousie University, Halifax, NS Canada; 3 Department of Community Health and Epidemiology, Dalhousie University, Halifax, NS Canada; 4 Heart and Stroke Foundation of Nova Scotia Endowed Chair in Cardiovascular Outcomes Research, Halifax, NS Canada; 5 Research Institute of St Joes Hamilton, St. Joseph’s Healthcare, Hamilton, ON Canada; 6 Faculty of Health Sciences, University of Johannesburg, Johannesburg, South Africa; 7 Departments of Anesthesia/Pediatrics, McMaster University, Hamilton, ON Canada; 8 Biostatistics Unit, The Research Institute, St Joseph’s Healthcare, Hamilton, ON Canada; 9 Population Health Research Institute (PHRI), Hamilton Health Sciences, McMaster University, Hamilton, ON Canada; 10 Sydney Primary Care Medical Clinic, Sydney, NS Canada; 11 Strive Health Management Consulting Ltd, Halifax, NS Canada; 12 QEII Health Sciences Centre, Nova Scotia Health Authority, Halifax, NS Canada; 13 Medical Affairs – Cardiovascular Medicine, Bayer Inc, Mississauga, ON Canada; 14 Medical & Scientific Affairs, Bayer Inc, Mississauga, ON Canada; 15 Centre for Health Economics and Policy Analysis, McMaster University, Hamilton, ON Canada

**Keywords:** Atrial fibrillation, Costs, Resource utilization, Decision support tool, Randomized controlled trial

## Abstract

**Background:**

IMPACT-AF is a prospective, randomized, cluster design trial comparing atrial fibrillation (AF) management with a computerized decision support system (CDS) to usual care (control) in the primary care setting of Nova Scotia, Canada. The objective of this analysis was to compare the resource use and costs between CDS and usual care groups.

**Methods:**

Case costing data, 12-month self-administered questionnaires, and monthly diaries from IMPACT-AF were used in this analysis. Descriptive statistics were used to compare costs and resource use between groups. All costs are presented in 2021 Canadian dollars and cover the 12-month period of participation in the study.

**Results:**

A total of 1,145 patients enrolled in the trial. Case costing data were available for 466 participants (41.1%), 12-month self-administered questionnaire data for 635 participants (56.0%) and monthly diary data for 223 participants (19.7%). Emergency department visits and hospitalizations comprised the most expensive component of AF care. Across all three datasets, there were no statistically significant differences in costs or resource use between CDS and usual care groups.

**Conclusions:**

Although there were no significant differences in resource use or costs among CDS and usual care groups in the IMPACT-AF trial, this study provides insight into the methodology and practical challenges of collecting economic data alongside a trial.

**Registration:**

Clinicaltrials.gov (registration number: NCT01927367, date of registration: 2013-08-20).

**Supplementary Information:**

The online version contains supplementary material available at 10.1186/s12911-023-02329-7.

## Introduction

Atrial fibrillation (AF) is the most common arrhythmia, affecting approximately 200,000 Canadians [1] Because of its increasing prevalence among an aging population, and the increased rates of acute care and hospitalization among affected patients, [2] the economic burden of AF is substantial. It has been estimated that AF accounts for 4.6% of Canadian acute care inpatient costs and 2.6% of all hospital admissions in Canada, rising to 5.6% of all admissions for people aged 50 years and older [3].

A review of 27 studies found the cost of AF to be substantial, at both the individual and healthcare system levels, reflecting patients’ need for resource-intensive (i.e., hospitalization and acute care) and long-term treatments (e.g., anticoagulation treatment) [4]. More specifically, acute care was identified as being the most costly component, followed by outpatient expenses, physician reimbursement and then medication-related costs [4]. The 2020 Canadian Cardiovascular Society (CCS) AF guidelines estimated that the annual direct cost of AF care for the healthcare system was $956 million (adjusted to 2020 Canadian dollars). The guidelines note that the indirect per-patient costs (e.g. due to work absences) have been estimated at $3,082 (adjusted to 2020 Canadian dollars) – but this costing information is from the United States [5].

The objective of this analysis was to evaluate whether an electronic clinical decision support (CDS) system designed to assist both providers and patients with evidence-based management strategies for AF could reduce resource use and costs for AF patients compared to the usual standard of care.

## Methods

### Trial design

This is a pre-specified analysis of secondary cost and resource use outcomes of the Integrated Management Program Advancing Community Treatment of Atrial Fibrillation (IMPACT-AF) study. IMACT-AF was a prospective, randomized, unblinded, cluster designed trial of a CDS system for the management of AF in primary care. The study protocol [6] and findings for the primary efficacy outcomes and secondary health-related quality of life (HRQoL) outcomes have been previously reported [6–8]. This study adheres to CONSORT guidelines [9, 10].

### Intervention

The CDS in the IMPACT-AF trial was a web-based software tool designed to support the management of AF patients in primary care. It offered best practice recommendations in regard to diagnostic assessment and treatment, per Canadian AF clinical guidelines, allowed surveillance of AF patients through a range of data sources (electronic laboratory results and patient-reported data), and prompted primary care providers to respond to critical alerts and trends. The system also had web-based education and support for providers and patients. Specific features of the CDS included:

- Translation of best-evidence to primary care providers (PCPs) through computerization of Canadian AF clinical guidelines;
- Enabling specific tasks such as auto-calculation of stroke and bleeding risk scores, monitoring of international normalized ratio (INR) and creatinine clearance with subsequent recommendations for medication dosing adjustment, assisting with optimal warfarin management via a dosing calculator, and providing recommendations for initiation and switching OACs based on patient clinical characteristics and ability to pay for medications;
- Providing a non-vitamin-K antagonist (NOAC) special authorization tool that aligns with the Nova Scotia Seniors Pharmacare exception status criteria and automatically prepopulates a drug coverage application form.

Additional information on the CDS is reported elsewhere [6].

### Participants

Primary care practices in the province of Nova Scotia, Canada were randomized 1:1 to CDS (intervention) and usual care (control) groups. Patients within each practice were recruited by their provider to participate. Inclusion criteria were: 18 years of age or older, having electrocardiographically confirmed AF or documentation of past diagnosis or management of AF in their medical record, and ability to communicate in English and provide informed consent. The only exclusion criterion was having a poor likelihood of surviving 12 months after enrollment.

### Data collection

Clinical, laboratory, and treatment data relevant to the AF management of each participating patient were collected through electronic and/or paper medical record review by trained study abstractors at baseline and 12 months. Three sources of costing and resource use data were collected as part of the study: case costing data, self-administered questionnaire (12 months), and monthly diaries. Each dataset was designed to evaluate different types of costs. A detailed description of each source of costing and resource use data is provided below.

#### Case costing data

The case costing data recorded public payer costs for AF-related hospitalizations and ER visits. Case costing data were provided by the Nova Scotia Health Authority (NSHA). As part of the case costing procedure, the NSHA documented all participant-incurred costs for emergency room (ER) visits and hospitalizations located in the Central Zone of the province of Nova Scotia, which includes the capital city of Halifax as well as rural communities located in the Eastern Shore and West Hants counties [11]. Approximately 40% of the provincial population resides in the Central Zone. Case costing data for other Nova Scotia health zones (Western, Eastern, Northern) were not available.

At 12-months, the IMPACT-AF study team reviewed each participant’s medical chart to determine whether there was an AF-related ER visit or unplanned cardiovascular hospitalization during the study period. A visit to the ER was classified as AF-related due to one of the following causes: acute coronary syndrome, presyncope/syncope, transient ischemic attack/stroke, atrial fibrillation, flutter, worsening congestive heart failure including pulmonary edema or dyspnea of cardiac origin. An unplanned cardiovascular hospitalization (admission with an overnight stay in hospital) was predefined as due to one of the following causes: acute coronary syndrome, presyncope / syncope, transient ischemic attack / stroke, atrial fibrillation, flutter, pulmonary embolism / deep vein thrombosis /systemic embolism, worsening congestive heart failure including pulmonary edema or dyspnea of cardiac origin. The IMPACT-AF study team shared this list of AF-related ER visits and unplanned cardiovascular hospitalizations with NSHA staff, who provided detailed costing data for each encounter.

The costs provided by the NSHA are categorized as direct medical and non-medical costs. Direct medical costs are defined as the expenditures associated with health care provided to patients including medication, laboratory and imaging tests, and health care professionals. Direct non-medical costs are the expenses required to support the delivery of care services including housekeeping, power, security, and administration (also known as overhead costs) [12].

#### Self-administered questionnaire

The self-administered questionnaire recorded patient’s reported AF-related resource use during the trial in addition to other information (e.g., sociodemographic characteristics and HRQoL). All patients were invited to complete a questionnaire (paper or electronic) 12-months after study entry. The questionnaire collected information about whether the patient had experienced any AF-related ER visits, cardiovascular hospitalizations, family physician visits and specialist visits (cardiologist, AF clinic/anticoagulation nurse, complementary health care professional, or internal medicine specialist) in the past 12 months. Patients were also asked if they had taken any AF-relevant medications (e.g., NOAC, St John’s Wort, non-steroidal anti-inflammatory (NSAIDS)), hired a paid caregiver or experienced any time lost from work due to their AF.

#### Monthly diaries

Patients were also asked to complete monthly diaries (paper or online) during the 12-month study period, which were designed to record more comprehensive data on healthcare encounters, out-of-pocket costs, and indirect cost (i.e., productivity loss) that they, family members or caregivers incurred as part of AF-related care. This includes family physician visits, walk-in and/or after-hours clinics, specialist visits, INR testing, emergency department visits, and hospitalizations. For each type of healthcare resource, the patient was instructed to record the monthly number of visits, time spent on visit(s), out-of-pocket visit expenses, distance travelled, and time missed from work. Patients were also invited to record the monthly time costs for informal caregivers, including the hours missed from work or time spent accompanying the patient to appointments or providing AF-related care.

The monthly diaries were meant to supplement the self-administered questionnaire by providing a more detailed picture of the resource use for each patient, such as the time, expenses and distance travelled to received AF-related care. They were designed with input from health economists and clinical experts to ensure that items aligned with healthcare services a typical AF patient would seek. It was anticipated that monthly data collection from the diaries would minimize recall bias compared to the 12-month self-administered questionnaire [13, 14]. Patients were able to call a toll-free number for support with completing the monthly diary or 12-month questionnaire.

### Statistical analysis

Descriptive statistics were used to summarize the sociodemographic and clinical characteristics of patients. Since the distribution of the resource use and costing data is highly skewed, we present both medians and means. Costs were collected in 2017 Canadian dollars according to the timelines of the study and converted to 2021 dollars using the Canadian Consumer Price Index (CPI). All costs cover the 12-month period of participation in the study. The estimates obtained from the monthly diaries were summed across the 12 months to calculate a total per-patient estimate for the 12-month follow-up period.

As cost and resource use were secondary outcomes of the IMPACT-AF trial, completion of the self-administered questionnaire and diaries was voluntary in nature and not a mandatory component of participation in the trial. As a result, some patients did not want to complete either but did provide consent for the study team to collect their healthcare data from medical records. This permitted the evaluation of the study’s primary outcome measure (presented in a previous manuscript). In total, 93 out of 1,145 participants (8%) chose this option (*n* = 46 in the CDS arm and *n* = 47 in the usual care arm).

Given the amount and nature of missing data, results are reported as complete case analyses. When the proportion of missing data is substantial (greater than 40%), imputation may not provide an unbiased, reliable estimate [15]. In such cases, as with the IMPACT-AF resource use and cost data, it is recommended that the observed data be used for the analyses. In addition to a high proportion of missing data, some of the missing data were non-random, such as the NSHA case costing information that were available for central zone patients only. As a result, each dataset (case costing, self-administered questionnaire, and monthly diaries) is treated as a distinct subgroup and presented separately. Within each dataset, T-tests were used to compare differences in mean costs and resource use between CDS and usual care groups while chi-square tests were used to compare proportions. Comparisons across datasets were analyzed by cross tabulating common variables.

The IMPACT-AF trial was powered to detect a statistically significant difference in relative risk reduction for the study’s primary efficacy endpoint (a composite of unplanned cardiovascular hospitalizations and AF-related emergency department visits) and not to detect any meaningful differences in resource use or costs between study arms. As such, the analyses presented in this paper are considered pre-specified secondary endpoints [6]. All analyses were conducted using SAS version 9.4 software (SAS Institute Inc). The trial was registered with ClinicalTrials.gov (registration number: NCT01927367, date of registration: 2013-08-20).

### Availability of data and materials

The data that support the findings of this study are available on request from the corresponding author, FX.

### Ethics

All methods were carried out in accordance with local guidelines and regulations. Ethics approval was provided by the Nova Scotia Health Authority Research Ethics Board. All participants provided written informed consent.

## Results

### Main findings

**Fig. 1:**
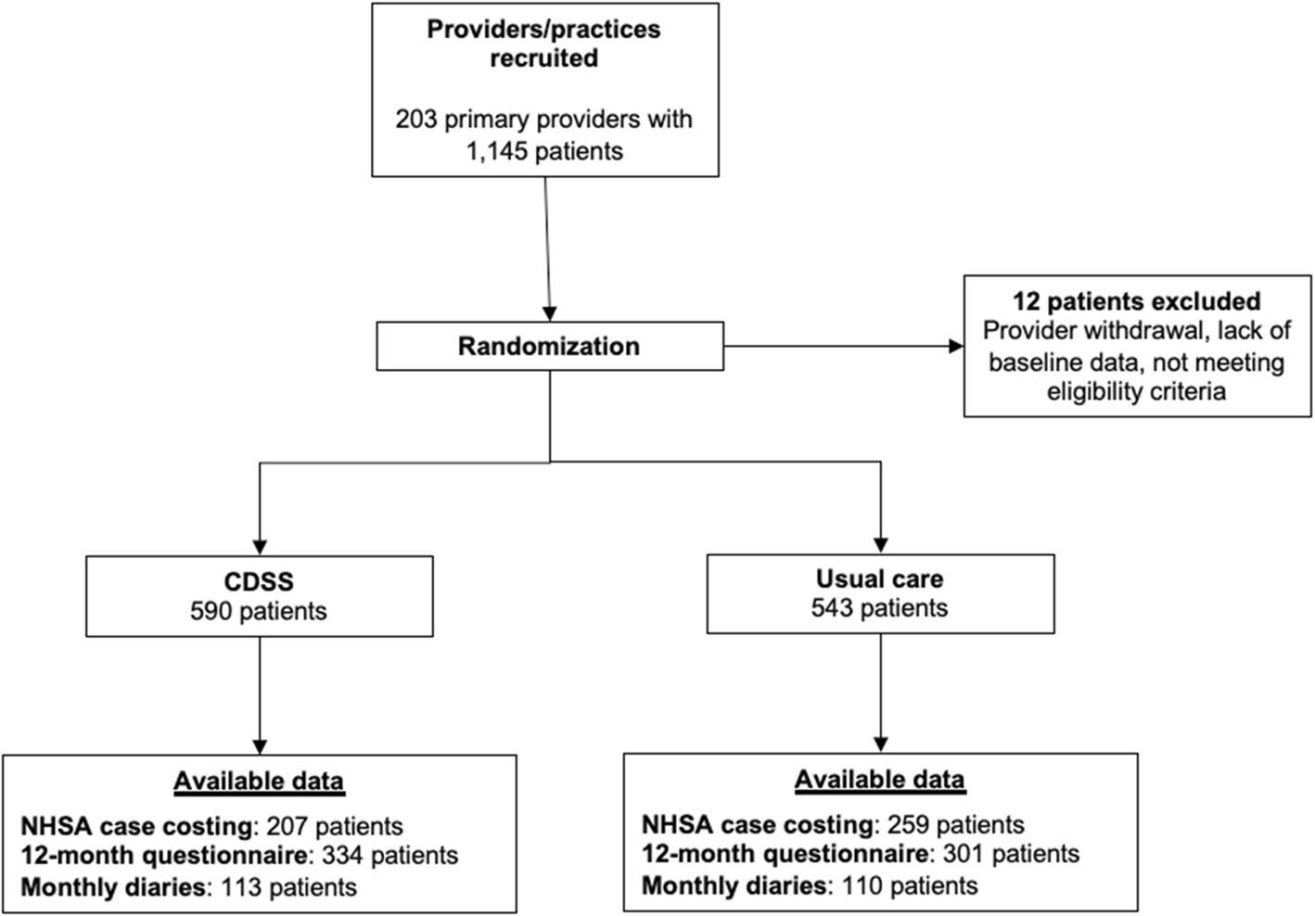
Patient flow

Between October 2014 and December 2016, 203 primary care providers and 1,145 of their patients were recruited, with equal and proportionate representation across Nova Scotia health zones [7]. Twelve patients were excluded because of provider withdrawal, lack of baseline data, or not meeting eligibility criteria, leaving 1133 patients for these analyses (*n* = 543 usual care, *n* = 590 CDS). NSHA case costing data were available for 466 participants (41.1%), 12-month self-administered questionnaire data for 635 participants (56.0%), and monthly diary data for 223 participants (19.7%). Figure 1 presents the patient flow.

Table 1 presents the baseline sociodemographic and clinical characteristics of patients according to each dataset (NSHA case costing, 12-month self-administered questionnaire, monthly diaries). Average age upon study entry ranged from 71 to 73 years across the three datasets and most participants were male. Unlike the 12-month questionnaire and monthly diaries, most participants in the case costing dataset lived in urban locations. However, 75 (16.1%) participants travelled from their residence in a rural location to a central zone hospital to receive care or received care at one of the rural facilities within the central zone (i.e., Hants Community Hospital, Twin Oaks Memorial Hospital or Musquodoboit Valley Memorial Hospital). Across all datasets, most participants reported that they were not working at the time of the study. Compared to the other datasets, a higher percentage of participants in the case costing data set had congestive heart failure, prior stroke, transient ischemic attack, systemic embolism, or vascular disease. While CHADS_2_andCHA_2_DS_2_-VASc scores were similar across all three datasets, they were slightly higher in the full sample.

**Table 1:** Baseline sociodemographic and clinical characteristics of patients according to dataset

	Full sample (*n* = 1133)		NSHA case costing (*n* = 466)		12-month questionnaire (*n* = 635)		Monthly diaries (*n* = 223)	
	n	(%)	n	(%)	n	(%)	n	(%)
**CDS**	590	(52.1)	207	(44.4)	334	(52.6)	113	(50.7)
**Sociodemographic characteristics**								
Age, years								
Mean (SD)	72.3	(10.0)	71.6	(10.5)	72.2	(8.9)	73.1	(8.2)
Male sex	701	(61.9)	303	(65.0)	396	(62.4)	124	(55.6)
Rural location	613	(54.1)	75	(16.1)	349	(55.0)	121	(54.3)
Highest level of completed education								
Some high school	165	(14.6)	58	(12.4)	121	(19.1)	45	(20.2)
Completed high school	161	(14.2)	63	(13.5)	105	(16.5)	44	(19.7)
College/Trade school	187	(16.5)	80	(17.2)	145	(22.8)	66	(29.6)
University undergraduate	105	(9.3)	51	(10.9)	79	(12.4)	27	(12.1)
University postgraduate	88	(7.8)	44	(9.4)	65	(10.2)	26	(11.7)
Not documented	393	(34.7)	170	(36.5)	190	(18.9)	15	(6.7)
Annual household income, Canadian dollars								
<$25 000	116	(10.2)	35	(7.5)	77	(12.1)	29	(13.0)
$25 000–$49 999	254	(22.4)	81	(17.4)	191	(30.1)	86	(38.6)
$50 000–$74 999	117	(10.3)	61	(13.1)	89	(14.0)	40	(17.9)
$75 000–$99 999	64	(5.7)	36	(7.7)	48	(7.6)	18	(8.1)
≥$100 000	57	(5.0)	33	(7.1)	40	(6.3)	10	(4.5)
Not Documented	525	(46.3)	220	(47.2)	190	(29.9)	40	(17.9)
Employment status								
Full time employed	62	(5.5)	30	(6.4)	37	(5.8)	9	(4.0)
Part time employed	36	(3.2)	15	(3.2)	26	(4.1)	8	(3.6)
Not working^a^	617	(54.5)	251	(53.9)	458	(72.1)	192	(86.1)
Not documented	418	(36.9)	170	(36.5)	114	(18.0)	14	(6.3)
**Clinical characteristics**								
Stroke risk, mean (SD)								
CHADS_2_	2.1	(1.3)	2.1	(1.4)	2.0	(1.3)	1.9	(1.3)
CHA_2_DS_2_-VAS_c_	3.7	(1.8)	3.6	(1.8)	3.5	(1.7)	3.5	(1.6)
Medical conditions								
Congestive heart failure	291	(25.7)	136	(29.2)	148	(23.3)	40	(17.9)
Hypertension	891	(78.6)	352	(75.5)	487	(76.7)	170	(76.2)
Diabetes	327	(28.9)	117	(25.1)	175	(27.6)	61	(27.4)
Prior stroke, transient ischemic attack, or systemic embolism	209	(18.5)	95	(20.4)	110	(17.3)	32	(14.3)
Vascular disease	398	(35.1)	179	(38.4)	212	(33.4)	69	(30.9)

Data on employment status, level of education and household income were obtained from the baseline questionnaire (paper or online), other data were obtained from the baseline case report form

*Abbreviations*: *CDS *Clinical decision support, *NSHA* Nova Scotia Health Authority, *SD* Standard deviation

^a^Includes retired, unemployed or homemaker

Table 2 presents the NSHA case costing data for emergency room visits and hospitalizations during the 12-month follow-up period. There were no statistically significant differences between CDS and usual care groups for type of encounter (ER visit, hospitalization, ER visit followed by hospitalization) or cost (direct medical, direct non-medical, total direct cost). ER visits were the most frequent type of encounter and direct medical costs the largest cost component. While the percent of patients experiencing an ER visit or hospitalization were similar across groups, the associated costs were slightly elevated in the CDS group. Mean total per-patient direct costs were $1,433 and $1,307 within CDS and usual care groups, respectively.

**Table 2:** NSHA case costing data for emergency room visits and hospitalizations during 12-month follow-up (*n* = 466)

	CDS (*n* = 207)		Usual care (*n* = 259)		
	n	(%)	n	(%)	*P*-value
**Number of encounters**					
ER visit only	19	(9.2)	24	(9.3)	0.291
Hospitalization only	3	(1.4)	6	(2.3)	0.259
ER visit followed by hospitalization^a^	1	(0.5)	0	(0.0)	0.337
**Direct medical**					
Mean (SD)	$1,177	($7,358)	$1,076	($4,698)	0.856
Median (Q1, Q3)	$0	($0, $0)	$0	($0, $0)	
**Direct non-medical**					
Mean (SD)	$354	($1,633)	$231	($1,069)	0.851
Median (Q1, Q3)	$0	($0, $0)	$0	($0, $0)	
**Total direct costs**					
Mean (SD)	$1,433	($8,984)	$1,307	($5,752)	0.856
Median (Q1,Q3)	$0	($0, $0)	$0	($0, $0)	

*Abbreviations*: *SD *Standard deviation, *Q1 *First quartile, *Q3 *Third quartile, *CDS* Clinical decision support, *ER *Emergency room

^a^Includes a participant who experienced an ER visit and hospitalization during a single recorded encounter (disaggregated data are not available)

Table 3 presents the resource use reported by patients in the 12-month questionnaire. For all variables, there were no statistically significant differences among CDS and usual care groups.

**Table 3:** Patient-reported resource use, expenses, and caregiver support in 12-month questionnaire (*n* = 635)

	CDS (*n* = 334)		Usual care (*n* = 301)		
	**n**	**(%)**	**n**	**(%)**	*P*-value
**Number of patients who reported at least one ER visit**	45	(13.5)	42	(14.0)	0.934
**Number of ER visits**					
Mean (SD)	0.3	(1.0)	0.4	(1.8)	0.536
Median (Q1, Q3)	0.0	(0.0, 0.0)	0.0	(0.0, 0.0)	
**Number of patients who reported at least one hospitalization**	22	(6.6)	21	(7.0)	0.763
**Number of times admitted to hospital**					
Mean (SD)	0.1	(0.4)	0.1	(0.3)	0.799
Median (Q1, Q3)	0.0	(0.0, 0.0)	0.0	(0.0, 0.0)	
**Number of patients who reported at least one family physician visit**	91	(27.2)	101	(33.6)	0.212
**Number of times visited family physician**					
Mean (SD)	0.9	(2.2)	1.1	(2.6)	0.168
Median (Q1, Q3)	0.0	(0.0, 0.0)	0.0	(0.0, 0.0)	
**Number of patients who reported at least one specialist visit**	158	(47.3)	141	(46.8)	0.756
**Type of specialist**					
AF clinic visit/Anticoagulation nurse	9	(2.7)	7	(2.3)	0.816
Cardiologist	121	(36.2)	105	(34.9)	0.792
Internal medicine	43	(12.9)	48	(15.9)	0.476
Complementary Health	6	(1.8)	1	(0.3)	0.265
**Number of patients with at least one NOAC medication**	136	(40.7)	128	(42.5)	0.407
**Number of patients with NOAC coverage**					
Nova Scotia Pharmacare	41	(30.1)	38	(29.7)	0.894
Private Insurance	68	(50.0)	68	(53.1)	0.494
Self	27	(19.9)	18	(14.8)	0.390
Not documented	0	(0.0)	4	(2.3)	
**Number of patients who reported use of other medications**					
St John’s Wort	2	(0.7)	1	(0.3)	0.632
Aspirin	53	(15.9)	38	(12.6)	0.582
Voltaren	29	(8.7)	24	(8.0)	0.643
Naproxen	2	(0.7)	1	(0.3)	0.633
Ibuprofen	6	(1.8)	4	(1.3)	0.650
**Number of patients who reported work loss due to AF**	8	(2.4)	10	(3.3)	0.661
**Number of patients who reported hiring a paid caregiver**	5	(1.5)	4	(1.3)	0.898

*Abbreviations*: *AF *Atrial fibrillation, *SD *Standard deviation, *Q1 *First quartile, *Q3 *Third quartile, *ER *Emergency room, *NOAC *Novel oral anticoagulant, *CDS* Clinical decision support

NOAC medications include Apixaban (Eliquis), Dabigatran (Pradaxa), Rivaroxaban (Xarelto), Edoxaban (Savaysa)

With regard to type of resource, patients in both CDS and usual care groups reported more ER visits than hospitalizations, which is in line with the case costing data presented in Table 2. More patients reported seeing a specialist for their AF (*n* = 158 CDS group, *n* = 141 usual care group) than a GP (*n* = 91 CDS group, *n* = 101 usual care group). Cardiologist was the most common type of specialist consulted for AF. With regard to medication, 136 (40.7%) participants in the CDS group and 128 (42.5%) participants in the usual care group reported using a NOAC. For these medications, private insurance was the most common form of medication coverage, followed by Nova Scotia’s Pharmacare Program. Few participants reported time lost from work (n = 8 CDS group, *n* = 10 usual care group) or hiring a paid caregiver (*n* = 5 CDS group, *n* = 4 usual care group) due to their AF.

More detailed information on the resource use and costs reported by patients was collected via the monthly diaries and is available as Supplementary material. In the diaries, INR testing was the category with the most intensive resource use for participants for both CDS and usual care groups. During the 12-month study period, participants reported an average of 6.30 and 6.90 INR visits among CDS and usual care groups, respectively. The second most common AF-related resource use in the diaries was family physician visits. Regardless of the type of resource, participants in both groups reported minimal expenses, time spent on visits and time missed from work associated with each AF-related encounter. During the 12-month study period, the average distance traveled was highest for INR testing (mean = 112.23 km CDS group, mean = 129.86 km usual care group), followed by family physician visits (mean = 64.24 km CDS group, mean = 51.72 km usual care group). The shortest distance traveled for both CDS and usual care groups was for walk-in clinic visits. The average time that informal caregivers reported spending on caring activities for AF patients over the 12 months of follow-up was 2.20 hours and 12.72 hours in the CDS and usual care groups, respectively. For all variables in the diaries, there were no statistically significant differences among CDS and usual care groups. 

### Sensitivity analyses

We conducted a sensitivity analysis to explore differences in resource use and costs between participants living in rural and urban locations. There were statistically significant differences between the two groups for the mean time missed from work for GP visits (*p*-value = 0.0084), time missed from work for INR visits (*p*-value = 0.0354), number of specialist visits (*p*-value = 0.0010), distance travelled for walk-in visits (*p*-value = 0.0261), and distance travelled for GP visits (*p*-value = 0.0285).

## Discussion

This study evaluated the resource use and costs associated with an electronic clinical decision support system for AF patients in Nova Scotia, Canada. During the 12-month follow-up period, there were no statistically significant differences in costs or resource use between CDS and usual care groups. Nevertheless, the data obtained from this study provide important insight into the management of AF in a single payer system at the primary care level.

Similar to other costing studies among the AF patient population, ER visits and hospitalizations comprised the expensive component of AF care [4] And, like these studies, the distribution of costs and resource use among patients in the IMPACT-AF trial suggests that there was a small percent of high-cost users [4]. However, unlike other costing studies, cost estimates were lower in the trial. A review of 27 estimates found that the mean annual direct cost for AF in Canada was $5,335 in 2010 CAD [4]. In contrast, the mean direct costs were $1,363 for IMPACT-AF participants. In addition, a US-based costing study found that the annual indirect costs (e.g., days of work missed because of illness) were $3,082 (converted to 2020 CAD) higher for AF patients compared with those without AF [5, 16]. In our study, participants reported incurring minimal time lost from work (range from a mean of 0 hours for ER visits to 4.23 hours for hospitalizations) or out-of-pocket expenses related to their AF care (range from a mean of $1 for walk-in clinic visits to mean of $21 for INR visits). The minimal time lost from work is expected given that the average age of participants was 72.3 years. Among the 63 participants who completed the 12-month questionnaire and who were employed, 18 (28.6%) reported time lost from work.

There are several reasons why patients’ costs may have been lower in the IMPACT-AF trial than other studies on the economic costs of AF. The first reason is that Nova Scotia has a public payer system in which the costs for hospitalizations, ER visits, family physicians, specialists, lab tests and, often, medications are covered by the province. This would reduce the cost burden for patients. A second reason is that participants in the study were being treated by primary health care professionals. Patients whose AF required management by specialists or a hospital-based AF clinic, with higher attendant costs, were excluded from the trial. A third reason why costs could have been lower in the IMPACT-AF trial is how they were measured. In the 12-month questionnaire and monthly diaries, participants were asked to report ‘AF-related’ costs and resource use. While some guidance was provided on potential reasons for AF-related ER visits (e.g., a racing heart), patients had to determine themselves if the visit was for their AF. A patient, for example, might see their physician for a reason other than AF, which could be opportunely addressed at the same visit. These patients might not see the association when filling out the study materials, which could result in underreporting of the costs or resource use associated with managing their AF. In addition, participants were instructed to only record certain costs in the diaries, such as the money spent for parking or on services received. The diaries did not include gas or meal expenses. As a result, the true costs that participants incurred due to their AF would have been higher as the data obtained from the diaries did indicate that some participants were driving long distances to receive AF care. The recently validated Cost for Patients Questionnaire identified a range of costs to be considered for patients, including travel costs, parking fees, prescription drugs, care services, medical devices, household renovations, medical tests, paramedical services, caregivers, accommodation for travel as well as costs associated with lost productivity [17].

While out of pocket costs and time lost from work were minimal for most of the patients in our study, some reported driving long distances to receive care. Specifically, participants reported travelling a mean distance of 121 km for INR testing and 58 km for family physician visits. This speaks to the frequency of INR visits relative to other types of care evaluated as well as the accessibility of AF care in Nova Scotia. Fifty-four percent of participants in this study lived in a rural area, where access to labs and family physicians are limited. The fact that participants reported travelling the longest distance for INR testing is a consideration for why AF patients should receive a NOAC instead of warfarin as these medications don’t require lab monitoring. Much of the costs associated with warfarin monitoring is indirect; the province pays for the labs and tests, which are inexpensive at $3.85 per test. However, it is the patient who pays for the time, effort, and expense involved with getting to the lab appointments.

Limitations of this study include the low percentage of patients who completed the diary and 12-month questionnaire. Case costing data sourced from administrative records provided the most accurate estimates of resource use and costs, but they were only available for central zone hospitals. Thus, apart from 75 patients (16.5%) who travelled from their residence in a rural location to a central zone hospital to receive care or received care at one of the rural facilities within the central zone, the case costing dataset excluded the majority of study participants from such districts (*n* = 613, 54.1%). The implication is that, depending on the geographic location, AF patients could have fundamentally different resource use and/or costs. A sensitivity analysis found statistically significant differences between rural and urban groups for the mean time missed from work for GP visits (*p*-value = 0.0084), time missed from work for INR visits (*p*-value = 0.0354), number of specialist visits (*p*-value = 0.0010), distance travelled for walk-in visits (*p*-value = 0.0261), and distance travelled for GP visits (*p*-value = 0.0285)

In addition to covering a limited group of participants, the case costing dataset provided information on ER visit and hospitalization costs only. To supplement this dataset, participants were asked to complete a monthly diary and 12-month questionnaire. The use of monthly diaries enabled a detailed record of the resources used and costs incurred from the patient perspective. Moreover, since monthly diaries collect information prospectively over a period of time, there is less recall error compared to a retrospective 12-month questionnaire [18]. Whereas, diaries are burdensome to complete and often have lower response rates than questionnaires [19], collecting both types of cost data in clinical trials is always challenging and a high percent of missing data is common [20]. This is one of the reasons why the research team opted to collect data from multiple sources. In this study, 19.7% of participants completed the monthly diaries compared to 56.0% for the 12-month questionnaires, resulting in a high amount of missing data. This could introduce a selection bias, where those that answered the survey may have different opinions or resource use than those that did not. Nevertheless, where patient information was available, a strength of our study is the use of multiple data sources to understand the broad spectrum of AF-related costs for patients and the healthcare system.

Another limitation of the study was the fact that only 3 in 4 providers in the CDS arm completed their training on how to use the CDS tool [7]. It was also suspected that only a proportion of those trained providers used the tool regularly due to implementation issues, including slow internet and operating speeds, lack of integration with key health datasets as well as the need for data entry and double login among other things [21]. This could explain the lack of any significant differences in costs and resource use between the CDS and usual care groups.

## Conclusion

Despite the lack of any significant differences in resource use or costs among CDS and usual care groups in the IMPACT-AF trial, this study provides unique insight into the economic burden of AF among patients treated in the primary care setting. While much of the AF literature reports on the management of AF by cardiologists or other specialists [22], AF is a common cardiac arrhythmia that primary care providers frequently manage. Understanding the full spectrum of AF-related resource use and costs can contribute to the development of new approaches for care delivery in the face of an increasing prevalence of AF in an aging population with a relatively shrinking number of providers. 

### Supplementary Information

**Additional file 1: Table s1.** Patient-reported resource use, expenses, and caregiver support in monthly diaries for 12 months (n = 223)

## Data Availability

The data that support the findings of this study are available on request from the corresponding author, FX.
