# Supplementary material for: Resource use and cost associated with computerized decision support system and usual care in managing patients with atrial fibrillation: analysis of IMPACT-AF randomized trial data
Source: BMC Med Inform Decis Mak. 2023 Oct 18;23:228. doi: 10.1186/s12911-023-02329-7 (PMC10585905; doi:10.1186/s12911-023-02329-7)
Supplement: Supplementary file 1 — Additional file 1: Table s1. Patient-reported resource use, expenses, and caregiver support in monthly diaries for 12 months (n = 223) [file 12911_2023_2329_MOESM1_ESM.docx]

**Supplementary material**

**table s1. Patient-reported resource use, expenses, and caregiver support in monthly diaries for 12 months (n = 223)**

|  | **CDS**  **(n = 113)** | | **Usual care**  **(n = 110)** | | P-value |
| --- | --- | --- | --- | --- | --- |
| **Family Physician** |  |  |  |  |  |
| Number of visits |  |  |  |  |  |
| Mean (SD) | 2.89 | (4.44) | 2.68 | (4.51) | 0.724 |
| Median (Q1, Q3) | 1.00 | (0.00, 4.00) | 1.00 | (0.00, 3.00) |  |
|  |  |  |  |  |  |
| Time spent on visits, hour |  |  |  |  |  |
| Mean (SD) | 3.60 | (6.77) | 3.57 | (7.71) | 0.821 |
| Median (Q1, Q3) | 1.00 | (0.00, 5.00) | 1.00 | (0.00, 4.00) |  |
|  |  |  |  |  |  |
| Visit expenses, $ |  |  |  |  |  |
| Mean (SD) | 2.35 | (9.34) | 4.86 | (28.09) | 0.393 |
| Median (Q1, Q3) | 0.00 | (0.00, 0.00) | 0.00 | (0.00, 0.00) |  |
|  |  |  |  |  |  |
| Distance travelled, km |  |  |  |  |  |
| Mean (SD) | 64.24 | (139.56) | 51.72 | (131.28) | 0.613 |
| Median (Q1, Q3) | 1.50 | (0.00, 46.00) | 6.00 | (0.00, 41.00) |  |
|  |  |  |  |  |  |
| Time missed from work, hour |  |  |  |  |  |
| Mean (SD) | 0.14 | (0.94) | 0.27 | (1.03) | 0.412 |
| Median (Q1, Q3) | 0.00 | (0.00, 0.00) | 0.00 | (0.00, 0.00) |  |
|  |  |  |  |  |  |
| **Walk-in/After-hours Clinic** |  |  |  |  |  |
| Number of visits |  |  |  |  |  |
| Mean (SD) | 0.33 | (1.88) | 0.15 | (0.59) | 0.354 |
| Median (Q1, Q3) | 0.00 | (0.00, 0.00) | 0.00 | (0.00, 0.00) |  |
|  |  |  |  |  |  |
| Time spent on visits, hour |  |  |  |  |  |
| Mean (SD) | 0.51 | (2.32) | 0.32 | (1.34) | 0.475 |
| Median (Q1, Q3) | 0.00 | (0.00, 0.00) | 0.00 | (0.00, 0.00) |  |
|  |  |  |  |  |  |
| Visit expenses, $ |  |  |  |  |  |
| Mean (SD) | 0.44 | (2.62) | 1.33 | (11.63) | 0.497 |
| Median (Q1, Q3) | 0.00 | (0.00, 0.00) | 0.00 | (0.00, 0.00) |  |
|  |  |  |  |  |  |
| Distance travelled, km |  |  |  |  |  |
| Mean (SD) | 7.49 | (32.55) | 3.97 | (20.52) | 0.339 |
| Median (Q1, Q3) | 0.00 | (0.00, 0.00) | 0.00 | (0.00, 0.00) |  |
|  |  |  |  |  |  |
| Time missed from work, hour |  |  |  |  |  |
| Mean (SD) | 0.11 | (1.03) | 0.01 | (0.11) | 0.374 |
| Median (Q1, Q3) | 0.00 | (0.00, 0.00) | 0.00 | (0.00, 0.00) |  |
|  |  |  |  |  |  |
| **Specialist** |  |  |  |  |  |
| Number of visits |  |  |  |  |  |
| Mean (SD) | 0.96 | (1.86) | 1.21 | (2.34) | 0.389 |
| Median (Q1, Q3) | 0.00 | (0.00, 1.00) | 0.00 | (0.00, 2.00) |  |
|  |  |  |  |  |  |
| Time spent on visits, hour |  |  |  |  |  |
| Mean (SD) | 1.89 | (4.47) | 2.91 | (8.87) | 0.390 |
| Median (Q1, Q3) | 0.00 | (0.00, 1.00) | 0.00 | (0.00, 1.50) |  |
|  |  |  |  |  |  |
| Visit expenses, $ |  |  |  |  |  |
| Mean (SD) | 5.83 | (19.36) | 7.88 | (45.90) | 0.681 |
| Median (Q1, Q3) | 0.00 | (0.00, 3.27) | 0.00 | (0.00, 2.73) |  |
|  |  |  |  |  |  |
| Distance travelled, km |  |  |  |  |  |
| Mean (SD) | 37.98 | (120.68) | 58.26 | (191.52) | 0.349 |
| Median (Q1, Q3) | 0.00 | (0.00, 22.40) | 0.00 | (0.00, 20.00) |  |
|  |  |  |  |  |  |
| Time missed from work, hour |  |  |  |  |  |
| Mean (SD) | 0.03 | (0.26) | 0.61 | (4.86) | 0.264 |
| Median (Q1, Q3) | 0.00 | (0.00, 1.00) | 0.00 | (0.00, 0.00) |  |
|  |  |  |  |  |  |
| **INR Testing** |  |  |  |  |  |
| Number of visits |  |  |  |  |  |
| Mean (SD) | 6.30 | (7.53) | 6.90 | (8.75) | 0.581 |
| Median (Q1, Q3) | 2.00 | (0.00, 12.00) | 2.50 | (0.00, 12.00) |  |
|  |  |  |  |  |  |
| Time spent on visits, hour |  |  |  |  |  |
| Mean (SD) | 7.96 | (15.15) | 8.27 | (25.33) | 0.914 |
| Median (Q1, Q3) | 1.00 | (0.00, 10.50) | 1.00 | (0.00, 10.00) |  |
|  |  |  |  |  |  |
| Visit expenses, $ |  |  |  |  |  |
| Mean (SD) | 21.44 | (55.57) | 19.66 | (51.82) | 0.806 |
| Median (Q1, Q3) | 0.00 | (0.00, 22.92) | 0.00 | (0.00, 14.19) |  |
|  |  |  |  |  |  |
| Distance travelled, km |  |  |  |  |  |
| Mean (SD) | 112.23 | (242.14) | 129.86 | (334.23) | 0.653 |
| Median (Q1, Q3) | 0.00 | (0.00, 125.00) | 2.75 | (0.00, 96.50) |  |
|  |  |  |  |  |  |
| Time missed from work, hour |  |  |  |  |  |
| Mean (SD) | 0.07 | (0.46) | 0.43 | (2.04) | 0.073 |
| Median (Q1, Q3) | 0.00 | (0.00, 0.00) | 0.00 | (0.00, 0.00) |  |
|  |  |  |  |  |  |
| **Emergency Department** |  |  |  |  |  |
| Number of visits |  |  |  |  |  |
| Mean (SD) | 0.52 | (1.32) | 0.32 | (0.93) | 0.183 |
| Median (Q1, Q3) | 0.00 | (0.00, 0.00) | 0.00 | (0.00, 0.00) |  |
|  |  |  |  |  |  |
| Time spent on visits, hour |  |  |  |  |  |
| Mean (SD) | 4.32 | (16.15) | 1.31 | (3.39) | 0.074 |
| Median (Q1, Q3) | 0.00 | (0.00, 0.00) | 0.00 | (0.00, 0.00) |  |
|  |  |  |  |  |  |
| Visit expenses, $ |  |  |  |  |  |
| Mean (SD) | 7.76 | (42.36) | 4.14 | (23.36) | 0.438 |
| Median (Q1, Q3) | 0.00 | (0.00, 0.00) | 0.00 | (0.00, 0.00) |  |
|  |  |  |  |  |  |
| Distance travelled, km |  |  |  |  |  |
| Mean (SD) | 9.54 | (34.28) | 7.83 | (45.85) | 0.708 |
| Median (Q1, Q3) | 0.00 | (0.00, 0.00) | 0.00 | (0.00, 0.00) |  |
|  |  |  |  |  |  |
| Time missed from work, hour |  |  |  |  |  |
| Mean (SD) | 0.29 | (2.49) | 0.00 | (0.00) | 0.252 |
| Median (Q1, Q3) | 0.00 | (0.00, 0.00) | 0.00 | (0.00, 0.00) |  |
|  |  |  |  |  |  |
| **Hospitalization** |  |  |  |  |  |
| Number of visits |  |  |  |  |  |
| Mean (SD) | 0.87 | (7.90) | 0.15 | (0.57) | 0.335 |
| Median (Q1, Q3) | 0.00 | (0.00, 0.00) | 0.00 | (0.00, 0.00) |  |
|  |  |  |  |  |  |
| Time spent on visits, hour |  |  |  |  |  |
| Mean (SD) | 1.72 | (9.52) | 5.79 | (52.12) | 0.482 |
| Median (Q1, Q3) | 0.00 | (0.00, 0.00) | 0.00 | (0.00, 0.00) |  |
|  |  |  |  |  |  |
| Visit expenses, $ |  |  |  |  |  |
| Mean (SD) | 9.00 | (69.86) | 0.82 | (5.12) | 0.250 |
| Median (Q1, Q3) | 0.00 | (0.00, 0.00) | 0.00 | (0.00, 0.00) |  |
|  |  |  |  |  |  |
| Distance travelled, km |  |  |  |  |  |
| Mean (SD) | 7.78 | (46.10) | 16.05 | (75.10) | 0.409 |
| Median (Q1, Q3) | 0.00 | (0.00, 0.00) | 0.00 | (0.00, 0.00) |  |
|  |  |  |  |  |  |
| Time missed from work, hour |  |  |  |  |  |
| Mean (SD) | 0.00 | (0.00) | 4.23 | (39.66) | 0.320 |
| Median (Q1, Q3) | 0.00 | (0.00, 0.00) | 0.00 | (0.00, 0.00) |  |
|  |  |  |  |  |  |
| **Informal caregiving** |  |  |  |  |  |
| Time spent accompanying to appointments, hour |  |  |  |  |  |
| Mean (SD) | 6.02 | (16.15) | 12.50 | (78.04) | 0.396 |
| Median (Q1, Q3) | 0.00 | (0.00, 2.00) | 0.00 | (0.00, 3.00) |  |
|  |  |  |  |  |  |
| Time spent on caring activities, hour |  |  |  |  |  |
| Mean (SD) | 2.20 | (16.15) | 12.72 | (76.38) | 0.160 |
| Median (Q1, Q3) | 0.00 | (0.00, 0.00) | 0.00 | (0.00, 0.00) |  |
|  |  |  |  |  |  |
| Time missed from work to accompany to appointments, hour |  |  |  |  |  |
| Mean (SD) | 1.23 | (6.96) | 4.19 | (34.40) | 0.377 |
| Median (Q1, Q3) | 0.00 | (0.00, 0.00) | 0.00 | (0.00, 0.00) |  |
|  |  |  |  |  |  |
| Time missed from work for caring activities, hour |  |  |  |  |  |
| Mean (SD) | 0.02 | (0.16) | 3.41 | (32.64) | 0.278 |
| Median (Q1, Q3) | 0.00 | (0.00, 0.00) | 0.00 | (0.00, 0.00) |  |
| Abbreviations: CDS, computerized decision support; SD, standard deviation; Q1, first quartile; Q3, third quartile; INR, international normalized ratio; km, kilometer | | | | | |
